# Impact of low bone mass and osteoporosis on lower limb alignment and knee function in high tibial osteotomy for varus knee osteoarthritis

**DOI:** 10.3389/fmed.2026.1922205

**Published:** 2026-09-30

**Authors:** Zhipeng Xue, Xiaomin Li, Ye Huang, Dafu Zhou, Yuanyi Hu, Xiangdong Tian

**Affiliations:** 1 Department of Minimally Invasive Joint, Third Affiliated Hospital, Beijing University of Chinese Medicine, Beijing, China; 2 Department of Orthopaedics, Wangjing Hospital, China Academy of Chinese Medical Sciences, Beijing, China

**Keywords:** high tibial osteotomy, knee function, low bone mass, lower limb alignment, osteoporosis, varus knee osteoarthritis

## Abstract

**Objective:**

To examine whether low bone mass or osteoporosis was associated with clinical and radiographic outcomes after high tibial osteotomy (HTO) in patients with varus knee osteoarthritis (KOA).

**Methods:**

This retrospective cohort included 123 patients with varus KOA who underwent medial open-wedge HTO between January 2019 and January 2024. Preoperative dual-energy x-ray absorptiometry classified 60 patients as having osteopenia or osteoporosis (T-score <−1.0; 42 with osteopenia and 18 with osteoporosis) and 63 as having normal bone mass (T-score ≥−1.0). Clinical outcomes included the Visual Analogue Scale (VAS), Western Ontario and McMaster Universities Osteoarthritis Index (WOMAC), and Lysholm score. Radiographic outcomes included the medial proximal tibial angle (MPTA), femorotibial angle (FTA), hip-knee-ankle angle (HKA), weight-bearing line ratio (WBLR), and posterior tibial slope (PTS). Outcomes were assessed preoperatively and during follow-up to 24 months. Because no primary endpoint or *a priori* sample-size calculation was prespecified, all analyses were considered exploratory.

**Results:**

Both groups improved from baseline in pain, function, and most alignment measures. At 24 months, no statistically significant between-group differences were detected in VAS, WOMAC, Lysholm, MPTA, FTA, HKA, WBLR, or PTS (all *P* > 0.05). The unadjusted 24-month mean differences (osteopenia/osteoporosis minus normal bone mass) were 0.07 points for VAS (95% CI, −0.10 to 0.24), 1.56 points for WOMAC (95% CI, −3.10 to 6.22), and −0.78 points for Lysholm (95% CI, −2.65 to 1.09). The excellent/good outcome rates were 88.33% and 90.48%, respectively, and complication rates were 5.00% and 3.17%, respectively. These estimates were imprecise for uncommon complications.

**Conclusions:**

In this retrospective cohort, low bone mass or osteoporosis was not significantly associated with inferior clinical or radiographic outcomes after HTO at 24 months. The absence of statistically significant differences should not be interpreted as evidence of equivalence. Treatment was completely confounded with bone-status group, and the study was not powered to exclude clinically meaningful differences or uncommon complications. Larger prospective studies with prespecified endpoints and standardized treatment protocols are warranted.

## 1 Introduction

Knee osteoarthritis (KOA) is a common degenerative joint disorder that causes pain, stiffness, and functional limitation (1). In varus KOA, abnormal mechanical alignment increases medial-compartment loading and may accelerate cartilage damage (2). Medial open-wedge high tibial osteotomy (HTO) redistributes load toward the lateral compartment and is an established joint-preserving procedure for appropriately selected patients (3). Bone quality is clinically relevant because maintenance of correction depends on osteotomy healing and stable fixation. Osteopenia and osteoporosis are common in the age group considered for HTO and may increase concern about delayed union, fixation failure, or loss of correction (4, 5). However, contemporary locking fixation and bone-health treatment may mitigate these risks, and published findings remain inconsistent (6, 7). We therefore examined whether preoperative bone-status category was associated with pain, knee function, alignment maintenance, osteotomy healing, and complications through 24 months after HTO.

## 2 Methods

### 2.1 Patient information

We retrospectively reviewed 123 patients with varus KOA who underwent HTO between January 2019 and January 2024. All patients met the clinical diagnostic criteria for varus KOA (8), had medial-compartment disease with lower-limb varus on radiography and computed tomography, and underwent preoperative dual-energy x-ray absorptiometry of the lumbar spine and femoral neck. Based on the recorded T-score, 60 patients were classified in the osteopenia/osteoporosis group, including 42 with osteopenia (−2.5 < T-score <−1.0) and 18 with osteoporosis (T-score ≤−2.5). The normal-bone-mass group comprised 63 patients with a T-score ≥−1.0. Of 156 screened patients, 33 were excluded and 123 formed the analytic cohort. All included patients underwent medial open-wedge HTO. Because this was a retrospective study without a prespecified protocol, no single primary endpoint was designated; the clinical and radiographic outcomes were analyzed as exploratory endpoints (Table 1).

**Table 1:** Comparison of baseline data between the two groups.

Index	Osteopenia/osteoporosis group (*n* = 60)	Normal-bone-mass group (*n* = 63)	Statistical value	*P* value
Gender (male/female)	26/34 (43.33/56.67)	28/35 (44.44/55.56)	*Χ*^2^ = 0.021	0.885
Age (year)	56.78 ± 5.32	55.92 ± 5.67	t = 0.821	0.413
Disease duration (year)	4.89 ± 1.67	4.65 ± 1.72	t = 0.723	0.471
Affected side (left/right)	31/29 (51.67/48.33)	33/30 (52.38/47.62)	*Χ*^2^ = 0.012	0.913
BMI (kg/m^2^)	26.34 ± 2.15	25.98 ± 2.31	t = 0.845	0.400
Bone mineral density T-value	−1.89 ± 0.56	−0.67 ± 0.23	t = 15.287	<0.001

### 2.2 Inclusion and exclusion criteria

Inclusion criteria: (1) Age 40-65 years, male or female; (2) Met the diagnostic criteria for varus KOA, with cartilage wear in the medial compartment of the knee joint classified as Kellgren-Lawrence grade II-III, and cartilage wear in the lateral compartment < Kellgren-Lawrence grade II; (3) Varus deformity of the lower limb, with medial proximal tibial angle (MPTA) < 85°; (4) Complete bone mineral density test data, meeting the grouping criteria; (5) Follow-up duration ≥24 months, with complete clinical and imaging data.

Exclusion criteria: (1) Concurrent other joint diseases such as rheumatoid arthritis or ankylosing spondylitis; (2) Injury to the cruciate ligaments or collateral ligaments of the knee joint, resulting in poor joint stability; (3) Surgical complications such as infection at the osteotomy site or nonunion; (4) Severe comorbidities involving the heart, liver, kidneys, or other organs, rendering the patient unable to tolerate surgery and follow-up; (5) Previous history of knee surgery.

### 2.3 Surgical technique

All surgical procedures were performed by the same experienced team of senior surgeons. Patients were placed in a supine position. After anesthesia was administered, arthroscopic debridement and exploration of the knee joint were performed first. The medial compartment, lateral compartment, intercondylar notch, and patellofemoral joint were systematically examined sequentially, and pathological tissues were treated accordingly based on the arthroscopic findings. Following this, the joint cavity was thoroughly irrigated with a large volume of normal saline, and the incision was sutured. Subsequently, the high tibial osteotomy was performed at a plane distal to the tibial tubercle. The osteotomy plane was planned intraoperatively, positioned at the junction of the distal one-third of the tibial tubercle, approximately 0.5 cm distal to the proximal tibiofibular joint, forming a 30° angle with the joint line. After confirming this plane using a C-arm fluoroscope, the osteotomy line was marked on the skin. The surgical incision was then determined: centering on the intersection point of the osteotomy line and a point 0.5 cm anterosuperior to the medial posterior border of the tibia, a 4 to 6 cm longitudinal incision was made. The skin, fascia, and pes anserinus were sequentially incised, and the superficial and deep medial collateral ligaments, as well as part of the proximal tibial periosteum, were elevated. Following the pre-marked osteotomy line, a Kirschner wire was inserted from the proximal tibia posteriosuperiorly (toward the fibular head). After reconfirming the correct osteotomy direction and position with C-arm fluoroscopy, the osteotomy was commenced. A large blunt retractor was placed posterior to the tibia to effectively protect the surrounding soft tissues and neurovascular structures. An oscillating saw was used to perform the precise osteotomy along the predetermined line, preserving approximately 1 cm of the lateral tibial cortex as a hinge. Subsequently, five holes were drilled along the osteotomy site using a Kirschner wire to weaken the lateral cortex. After completing the osteotomy, an assistant stabilized the proximal fragment while externally rotating the patient's foot until the first toe pointed 15° lateral to the neutral position. The surgeon, with one hand supporting the lateral aspect of the osteotomy site and the other holding the ankle, slowly applied a valgus force. Under C-arm fluoroscopic guidance, the angle formed between the line connecting the femoral condyles and the longitudinal axis of the fibula was confirmed to reach 93° after distraction. The opened gap was filled with allograft bone, and the osteotomy was securely fixed with a locking compression plate (Figure 1). After confirming satisfactory implant position with fluoroscopy, a drain was placed in the surgical area, the incision was sutured in layers, and a sterile dressing was applied.

**Figure 1:**
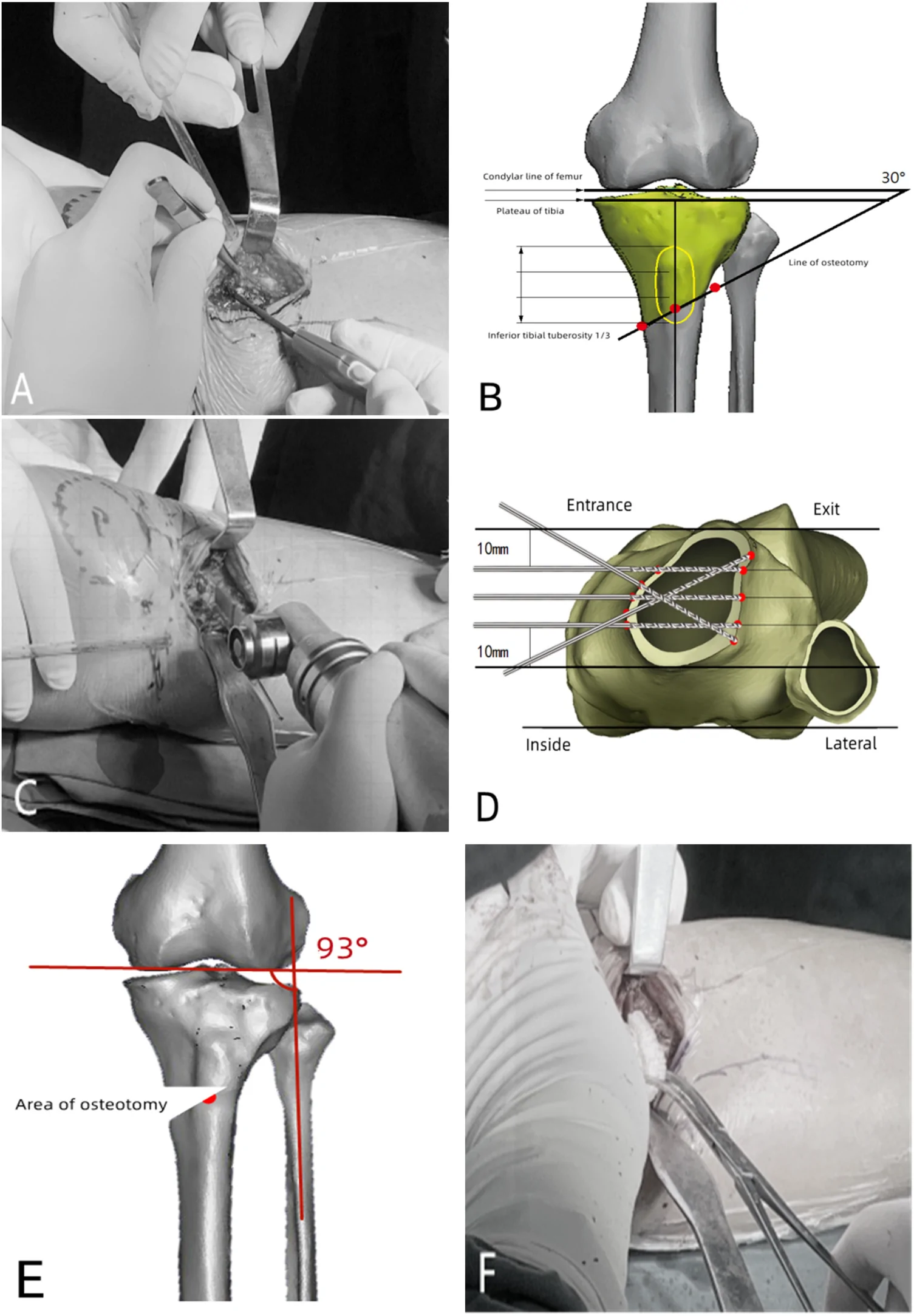

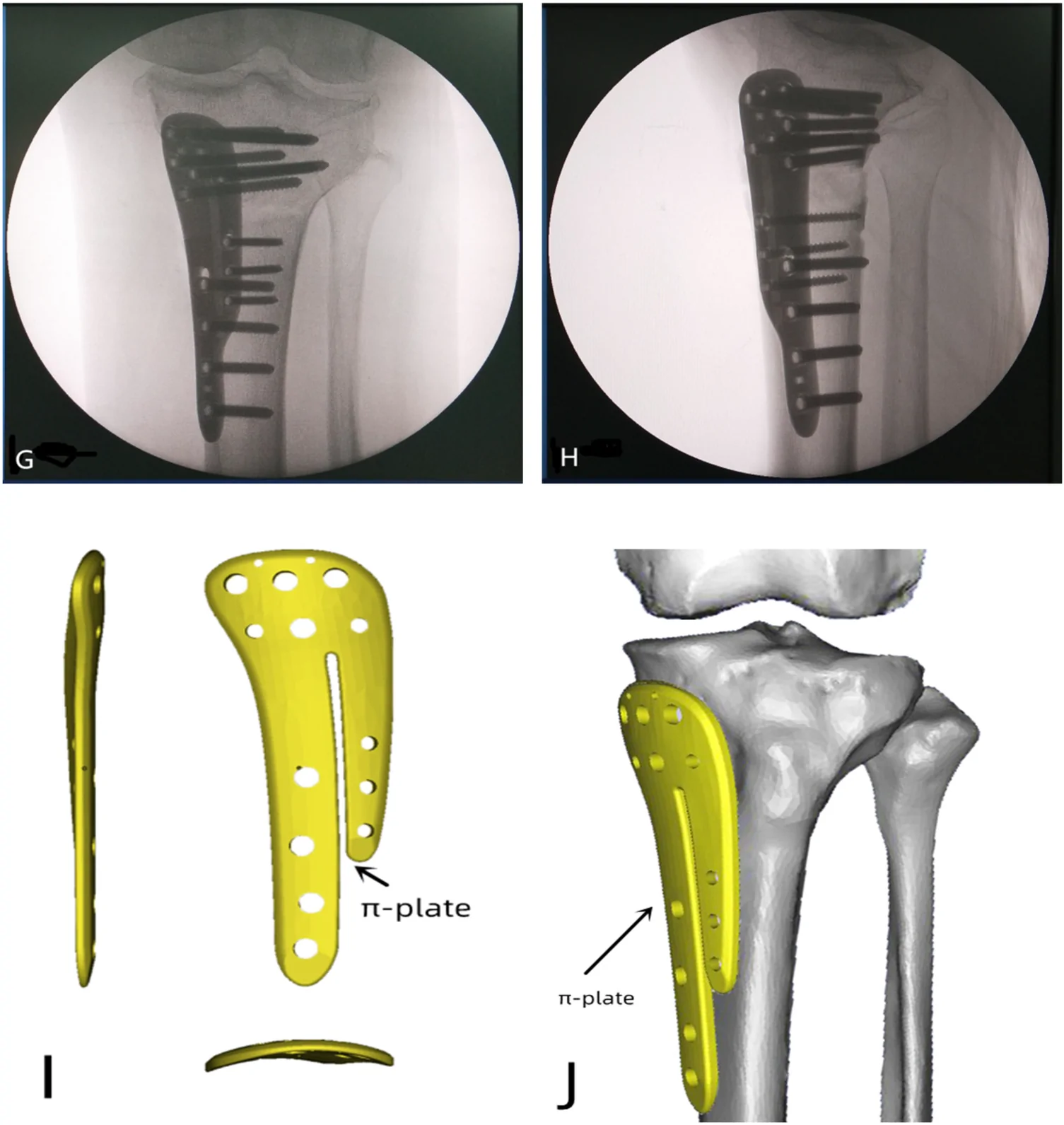
The pictures are made by Adobe photoshop 2021. HTO surgical roadmap **(A)** soft tissue stripping; **(B)** Kirschner wire positioning osteotomy line; **(C)** Oscillating saw osteotomy; **(D)** Weakening the lateral cortex operation; **(E)** Bone interception operation; **(F)** Allogeneic bone was implanted; **(G–J)** Locking plate fixation.

### 2.4 Postoperative management and follow-up

The postoperative management protocol was consistent for both groups: routine postoperative anti-infection, analgesic, and anti-edema treatments were administered. The low bone mass and osteoporosis group received additional anti-osteoporosis treatment (oral Calcium Carbonate and Vitamin D3 tablets, 0.6 g once daily; Alendronate Sodium tablets, 70 mg once weekly) for 12 months. On postoperative days 1–2, patients were instructed on ankle pump exercises and isometric quadriceps contractions. One week post-surgery, gradual knee flexion and extension functional training was initiated. At 4 weeks post-surgery, patients could begin partial weight-bearing ambulation with crutches. Based on the healing status of the osteotomy site, gradual progression to full weight-bearing was achieved by 8–12 weeks post-surgery. The final follow-up was completed at 24 months postoperatively.

Clinical scores were recorded preoperatively and at 6 and 24 months after surgery. Radiographic alignment was recorded preoperatively, immediately after correction, and at 24 months. The analytic cohort was restricted to patients with complete clinical and imaging data through 24 months; therefore, no included patient had missing values at the reported time points. This complete-case design may introduce selection bias and is acknowledged as a limitation.

### 2.5 Observation indicators

(1) Pain Score: Pain was assessed using the Visual Analogue Scale (VAS) (9), ranging from 0 to 10 points, where 0 indicates no pain and 10 indicates excruciating pain. A higher score suggests more severe knee pain.
(2) Knee Function Scores: Knee function was evaluated using the Western Ontario and McMaster Universities Osteoarthritis Index (WOMAC) (10) and the Lysholm Knee Scoring Scale (11). The WOMAC score encompasses three dimensions: pain (0–50 points), stiffness (0–20 points), and physical function (0–170 points), with a total score ranging from 0 to 240 points. A higher score indicates poorer knee function. The Lysholm score includes eight items such as swelling, pain, instability, and locking, with a total score ranging from 0 to 100 points. A higher score indicates better knee function.
(3) Lower Limb Alignment Parameters: Full-length standing radiographs of both lower limbs were used. MPTA was defined as the medial angle between the tibial mechanical axis and the tibial plateau. FTA was measured between the femoral and tibial axes, HKA between the femoral and tibial mechanical axes, and WBLR as the percentage position at which the weight-bearing line crossed the tibial plateau from medial to lateral. PTS was measured on the lateral radiograph relative to the proximal tibial reference axis. Alignment retention was summarized as the change between immediate correction and the 24-month measurement for MPTA, HKA, and WBLR. A formal intraobserver or interobserver reliability study was not available for these retrospective measurements.
(4) Surgical Efficacy: Surgical efficacy was evaluated at 24 months postoperatively based on the Lysholm score. The excellent and good rate was calculated as: (number of excellent + good cases)/total number of cases × 100%. The criteria were: Excellent: Lysholm score ≥90 points; Good: 80 points ≤ Lysholm score <90 points; Fair: 70 points ≤ Lysholm score< 80 points; Poor: Lysholm score <70 points.
(5) Osteotomy Healing, Alignment Maintenance and Internal Fixation-Related Evaluation Indicators: Combined with the clinical characteristics of HTO in patients with low bone mass and osteoporosis, targeted refined indicators were supplemented for core clinical concerns. ① Osteotomy healing index: The postoperative osteotomy healing time of each patient was accurately recorded (unit: month), and healing status was divided into normal healing (healing time ≤3 months) and delayed healing (healing time >3 months) according to imaging healing performance and clinical weight-bearing recovery, to evaluate the effect of bone mass status on osteotomy healing efficiency. ② Correction retention index: The postoperative loss of lower limb alignment correction was quantitatively analyzed to assess the long-term stability of surgical correction effect. ③ Implant and bone healing-related complications: Focused recording and statistical analysis of osteoporosis-specific adverse complications including osteotomy delayed union, nonunion, lateral hinge fracture, internal fixation loosening, and progressive long-term lower limb alignment offset were performed to evaluate internal fixation stability and bone healing quality in the two groups.
(6) Complications: The occurrence of complications during the treatment and follow-up period was recorded for both groups.

### 2.6 Statistical methods

Statistical analyses were performed using SPSS version 26.0. Continuous variables are reported as mean ± standard deviation, and categorical variables as number and percentage. Separate linear mixed-effects models were specified for clinical outcomes measured preoperatively and at 6 and 24 months and for radiographic outcomes measured preoperatively, immediately after correction, and at 24 months. Each model included group, time, and the group-by-time interaction as fixed effects, with patient identifier as a random intercept and an AR(1) covariance structure. Pairwise within-group and between-group comparisons were exploratory. Two-sided *P* values were reported without multiplicity adjustment and should therefore be interpreted as nominal. Unadjusted between-group mean differences and 95% confidence intervals were calculated for the 24-month outcomes. Exploratory multivariable linear regression assessed the association between bone-status group and 24-month outcomes after adjustment for available clinical and alignment covariates. Anti-osteoporosis treatment was not included in the same model as bone-status group because treatment assignment was completely determined by group, precluding separate estimation of their independent effects. No *a priori* sample-size calculation or formal power analysis was performed. Accordingly, non-significant findings were not interpreted as equivalence, and the low event counts limited inference about uncommon complications. A two-sided *P* value <0.05 was considered statistically significant.

## 3 Results

### 3.1 Comparison of baseline data between the two groups

Age, sex, disease duration, affected side, and body mass index did not differ significantly between groups (all *P* > 0.05). Bone mineral density T-score differed as expected from the exposure definition (*P* < 0.001). Baseline clinical scores and radiographic measures are reported with the corresponding outcome tables.

### 3.2 Comparison of VAS scores between the two groups

VAS decreased in both groups at 6 and 24 months compared with baseline. No statistically significant between-group difference was detected at any reported time point (Table 2).

**Table 2:** Comparison of VAS scores between the two groups (points, $\bar{x}\pm s$).

Index	Osteopenia/osteoporosis group (*n* = 60)	Normal-bone-mass group (*n* = 63)	*t* value	*P* value
Preoperative	7.23 ± 1.05	7.15 ± 1.12	0.365	0.816
6 months postoperative	2.12 ± 0.87	2.05 ± 0.91	0.823	0.563
24 months postoperative	1.34 ± 0.46	1.27 ± 0.49	0.899	0.611
F value	314.053	289.402		
*P* value	<0.001	<0.001		

### 3.3 Comparison of WOMAC scores between the two groups

WOMAC scores decreased in both groups at 6 and 24 months compared with baseline. No statistically significant between-group difference was detected at any reported time point (Table 3).

**Table 3:** Comparison of WOMAC scores between the two groups (points, $\bar{x}\pm s$).

Index	Osteopenia/osteoporosis group (*n* = 60)	Normal-bone-mass group (*n* = 63)	*t* value	*P* value
Preoperative	156.78 ± 23.45	154.32 ± 24.11	0.653	0.674
6 months postoperative	98.65 ± 18.72	96.89 ± 19.05	0.896	0.560
24 months postoperative	56.34 ± 12.89	54.78 ± 13.21	0.574	0.841
F value	734.635	785.564		
*P* value	<0.001	<0.001		

### 3.4 Comparison of Lysholm scores between the two groups

Lysholm scores increased in both groups at 6 and 24 months compared with baseline. No statistically significant between-group difference was detected at any reported time point (Table 4).

**Table 4:** Comparison of lysholm scores between the two groups of patients (points, $\bar{x}\pm s$).

Index	Osteopenia/osteoporosis group (*n* = 60)	Normal-bone-mass group (*n* = 63)	*t* value	*P* value
Preoperative	52.34 ± 8.76	53.12 ± 8.91	0.593	0.932
6 months postoperative	75.67 ± 9.23	76.89 ± 8.97	0.631	0.793
24 months postoperative	88.76 ± 5.34	89.54 ± 5.12	0.758	0.588
F value	529.643	594.565		
*P* value	<0.001	<0.001		

### 3.5 Comparison of lower limb alignment indicators between the two groups

#### 3.5.1 Comparison of MPTA between the two groups

MPTA increased after correction and remained improved at 24 months in both groups. No statistically significant between-group difference was detected at the reported time points (Table 5).

**Table 5:** Comparison of MPTA between the two groups of patients (°, $\bar{x}\pm s$).

Index	Osteopenia/osteoporosis group (*n* = 60)	Normal-bone-mass group (*n* = 63)	*t* value	*P* value
Preoperative	83.34 ± 2.15	83.56 ± 2.23	0.593	0.632
After intraoperative correction	93.65 ± 3.89	93.89 ± 3.76	0.631	0.593
24 months postoperative	92.93 ± 2.95	93.16 ± 2.87	0.758	0.588
F value	316.346	362.682		
*P* value	<0.001	<0.001		

### 3.6 Comparison of FTA between the two groups

FTA decreased after correction and remained improved at 24 months in both groups. No statistically significant between-group difference was detected at the reported time points (Table 6).

**Table 6:** Comparison of FTA between the two groups of patients (°, $\bar{x}\pm s$).

Index	Osteopenia/osteoporosis group (*n* = 60)	Normal-bone-mass group (*n* = 63)	*t* value	*P* value
Preoperative	179.18 ± 3.21	178.89 ± 3.15	0.363	0.892
After intraoperative correction	172.34 ± 2.15	172.72 ± 2.23	0.782	0.682
24 months postoperative	172.56 ± 2.54	172.14 ± 2.17	0.592	0.613
F value	391.421	382.375		
*P* value	<0.001	<0.001		

### 3.7 Comparison of HKA between the two groups

HKA increased after correction and remained improved at 24 months in both groups. No statistically significant between-group difference was detected at the reported time points (Table 7).

**Table 7:** Comparison of HKA between the two groups of patients (°, $\bar{x}\pm s$).

Index	Osteopenia/osteoporosis group (*n* = 60)	Normal-bone-mass group (*n* = 63)	*t* value	*P* value
Preoperative	172.67 ± 3.34	171.78 ± 1.29	0.813	0.593
After intraoperative correction	183.52 ± 2.76	183.78 ± 2.81	0.549	0.426
24 months postoperative	182.86 ± 1.82	183.02 ± 1.48	0.963	0.692
F value	395.323	372.573		
*P* value	<0.001	<0.001		

### 3.8 Comparison of WBLR between the two groups

WBLR increased after correction and remained improved at 24 months in both groups. No statistically significant between-group difference was detected at the reported time points (Table 8).

**Table 8:** Comparison of WBLR between the two groups of patients (%, $\bar{x}\pm s$).

Index	Osteopenia/osteoporosis group (*n* = 60)	Normal-bone-mass group (*n* = 63)	*t* value	*P* value
Preoperative	18.34 ± 5.67	17.32 ± 5.49	0.788	0.618
After intraoperative correction	63.45 ± 2.18	64.12 ± 2.98	0.565	0.639
24 months postoperative	62.89 ± 2.35	63.56 ± 2.21	0.981	0.561
F value	816.598	862.424		
*P* value	<0.001	<0.001		

### 3.9 Comparison of PTS between the two groups

PTS did not change significantly across the reported time points in either group, and no statistically significant between-group difference was detected (Table 9).

**Table 9:** Comparison of PTS between the two groups of patients (°, $\bar{x}\pm s$).

Index	Osteopenia/osteoporosis group (*n* = 60)	Normal-bone-mass group (*n* = 63)	*t* value	*P* value
Preoperative	10.34 ± 1.21	10.41 ± 1.19	0.312	0.835
After intraoperative correction	10.45 ± 1.17	10.52 ± 1.15	0.218	0.894
24 months postoperative	10.39 ± 1.23	10.45 ± 1.18	0.226	0.842
F value	31.572	28.317		
*P* value	0.425	0.511		

### 3.10 Comparison of surgical efficacy between the two groups at 24 months postoperatively

At 24 months, the excellent/good outcome rate was 88.33% (53/60) in the osteopenia/osteoporosis group and 90.48% (57/63) in the normal-bone-mass group. The unadjusted risk difference was −2.15 percentage points (95% CI, −13.0 to 8.7; *P* = 0.669). The confidence interval does not establish equivalence (Table 10).

**Table 10:** Comparison of surgical efficacy between the two groups at 24 months postoperatively [*n* (%)].

Group	*n*	Excellent	Good	Fair	Poor	Excellent/good rate
Osteopenia/osteoporosis group	60	38 (63.33)	15 (25.00)	5 (8.33)	2 (3.34)	53 (88.33)
Normal-bone-mass group	63	40 (63.49)	17 (26.98)	4 (6.35)	2 (3.17)	57 (90.48)
*χ*^2^ value						0.182
*P* value						0.669

### 3.11 Comparison of complications between the two groups

Complications occurred in 3 of 60 patients (5.00%) in the osteopenia/osteoporosis group and 2 of 63 patients (3.17%) in the normal-bone-mass group. The unadjusted risk difference was 1.83 percentage points (95% CI, −5.2 to 8.9; *P* = 0.620). The low event count precludes a reliable conclusion about equivalence or rare-event risk (Table 11).

**Table 11:** Comparison of complications between the two groups [*n* (%)].

Group	*n*	Delayed union at the osteotomy site	Knee stiffness	Internal fixation loosening	Complication rate
Osteopenia/osteoporosis group (*n* = 60)	60	2 (3.33)	1 (1.67)	0 (0.00)	3 (5.00)
Normal-bone-mass group (*n* = 63)	63	0 (0.00)	1 (1.59)	1 (1.59)	2 (3.17)
*χ*^2^ value					0.245
*P* value					0.620

### 3.12 Osteotomy healing, correction loss and internal fixation-related complication analysis

Osteotomy healing time and losses of HKA, MPTA, and WBLR correction were numerically greater in the osteopenia/osteoporosis group, but the corresponding comparisons were not statistically significant (all *P* > 0.05). No lateral hinge fracture occurred. Because event counts were small, these findings do not exclude clinically important differences in delayed healing, progressive alignment offset, or fixation loosening (Table 12).

**Table 12:** Osteotomy healing, correction loss and internal fixation-related complication analysis.

Indicators	Osteopenia/osteoporosis group (*n* = 60)	Normal-bone-mass group (*n* = 63)	*t*/*χ*^2^ value	*P* value
Osteotomy healing time (month, $\bar{x}\pm s$)	3.12 ± 0.85	2.85 ± 0.72	1.832	0.069
Delayed osteotomy healing [*n* (%)]	5 (8.33)	2 (3.17)	1.526	0.217
*Δ*HKA correction loss (°, $\bar{x}\pm s$)	1.12 ± 0.45	0.98 ± 0.42	1.795	0.075
*Δ*MPTA correction loss (°, $\bar{x}\pm s$)	0.72 ± 0.31	0.65 ± 0.28	1.326	0.187
*Δ*WBLR correction loss (%, $\bar{x}\pm s$)	1.52 ± 0.68	1.38 ± 0.62	1.185	0.238
Hinge fracture [*n* (%)]	0 (0.00)	0 (0.00)	–	–
Internal fixation loosening [*n* (%)]	0 (0.00)	1 (1.59)	0.962	0.327
Progressive limb alignment offset [*n* (%)]	1 (1.67)	0 (0.00)	1.059	0.303

### 3.13 Between-group effect estimates at 24 months

Unadjusted effect estimates at 24 months were calculated from the reported group means, standard deviations, and sample sizes (Table 13). Confidence intervals quantify precision and should not be interpreted as equivalence bounds.

**Table 13:** Unadjusted between-group effect estimates at 24 months.

Outcome	Osteopenia/osteoporosis	Normal bone mass	Effect estimate (95% CI)	*P* value
VAS score	1.34 ± 0.46	1.27 ± 0.49	Mean difference 0.07 (−0.10 to 0.24)	0.611
WOMAC score	56.34 ± 12.89	54.78 ± 13.21	Mean difference 1.56 (−3.10 to 6.22)	0.841
Lysholm score	88.76 ± 5.34	89.54 ± 5.12	Mean difference −0.78 (−2.65 to 1.09)	0.588
MPTA (degrees)	92.93 ± 2.95	93.16 ± 2.87	Mean difference −0.23 (−1.27 to 0.81)	0.588
FTA (degrees)	172.56 ± 2.54	172.14 ± 2.17	Mean difference 0.42 (−0.43 to 1.27)	0.613
HKA (degrees)	182.86 ± 1.82	183.02 ± 1.48	Mean difference −0.16 (−0.75 to 0.43)	0.692
WBLR (%)	62.89 ± 2.35	63.56 ± 2.21	Mean difference −0.67 (−1.49 to 0.15)	0.561
PTS (degrees)	10.39 ± 1.23	10.45 ± 1.18	Mean difference −0.06 (−0.49 to 0.37)	0.842
Excellent/good outcome	53/60 (88.33%)	57/63 (90.48%)	Risk difference −2.15 pp (−13.0 to 8.7)	0.669
Any complication	3/60 (5.00%)	2/63 (3.17%)	Risk difference 1.83 pp (−5.2 to 8.9)	0.620

### 3.14 Exploratory multivariable analysis

Exploratory multivariable linear regression estimates are summarized in Table 14. These models assess association, not causation. Anti-osteoporosis treatment could not be entered together with bone-status group because treatment was assigned to all patients in the osteopenia/osteoporosis group and to none in the normal-bone-mass group. The independent effects of treatment and bone status are therefore not identifiable in this cohort.

**Table 14:** Exploratory association of bone-status group With 24-month outcomes.

Outcome	Adjusted beta for bone-status group	95% CI	*P* value
VAS score	0.12	−0.31 to 0.55	0.584
WOMAC score	2.15	−4.78 to 9.08	0.542
Lysholm score	−0.89	−3.12 to 1.34	0.433
MPTA	0.23	−0.89 to 1.35	0.687
FTA	−0.18	−1.05 to 0.69	0.685
HKA	0.31	−0.72 to 1.34	0.554
WBLR	0.42	−0.96 to 1.80	0.551

### 3.15 Typical case

A 63-year-old woman with bilateral varus KOA and osteoporosis underwent right HTO, followed by left HTO after progression of contralateral symptoms. Serial radiographs through 2 years are shown as an illustrative case only and were not used for inferential comparison (Figure 2).

**Figure 2:**
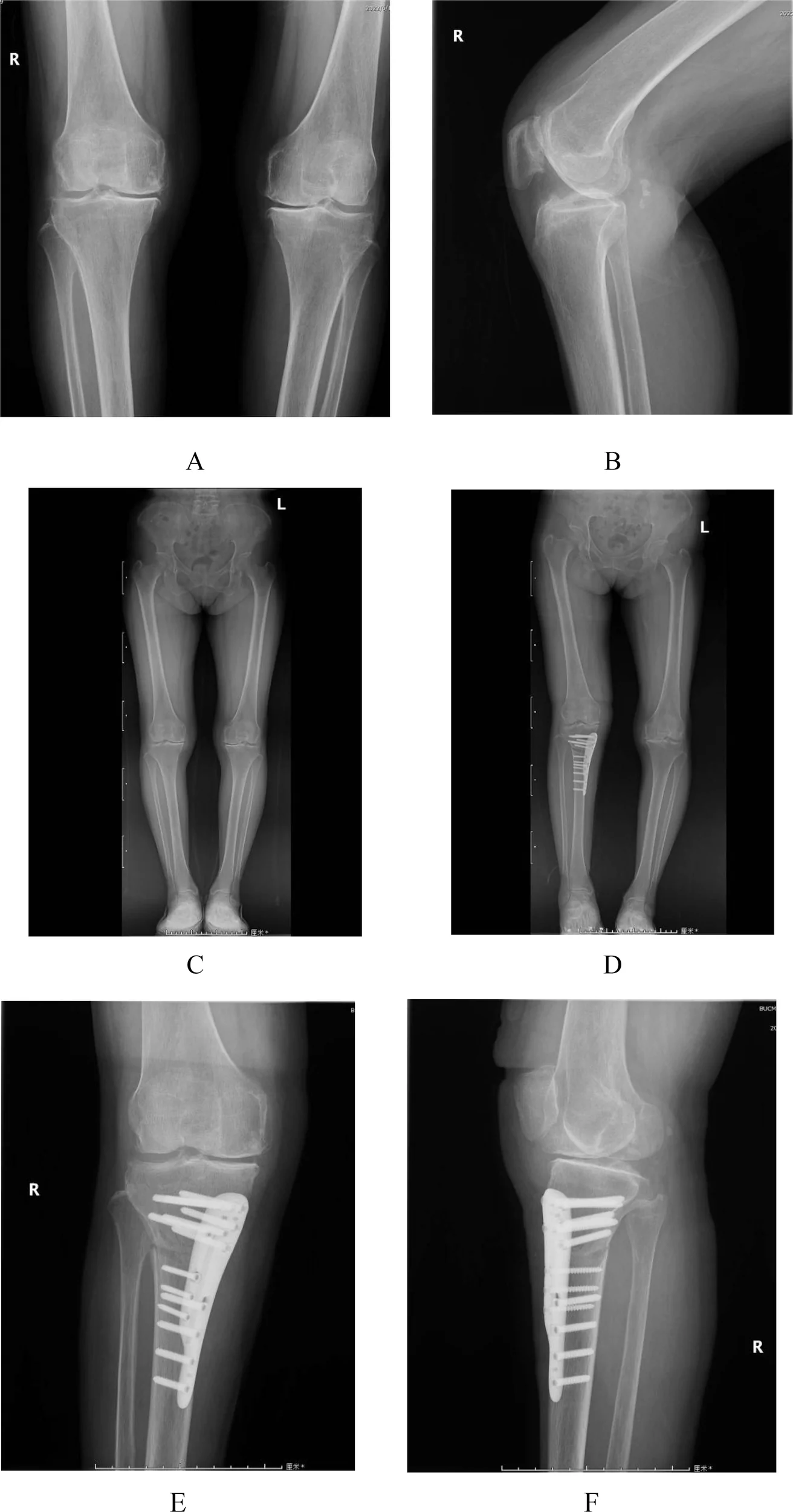

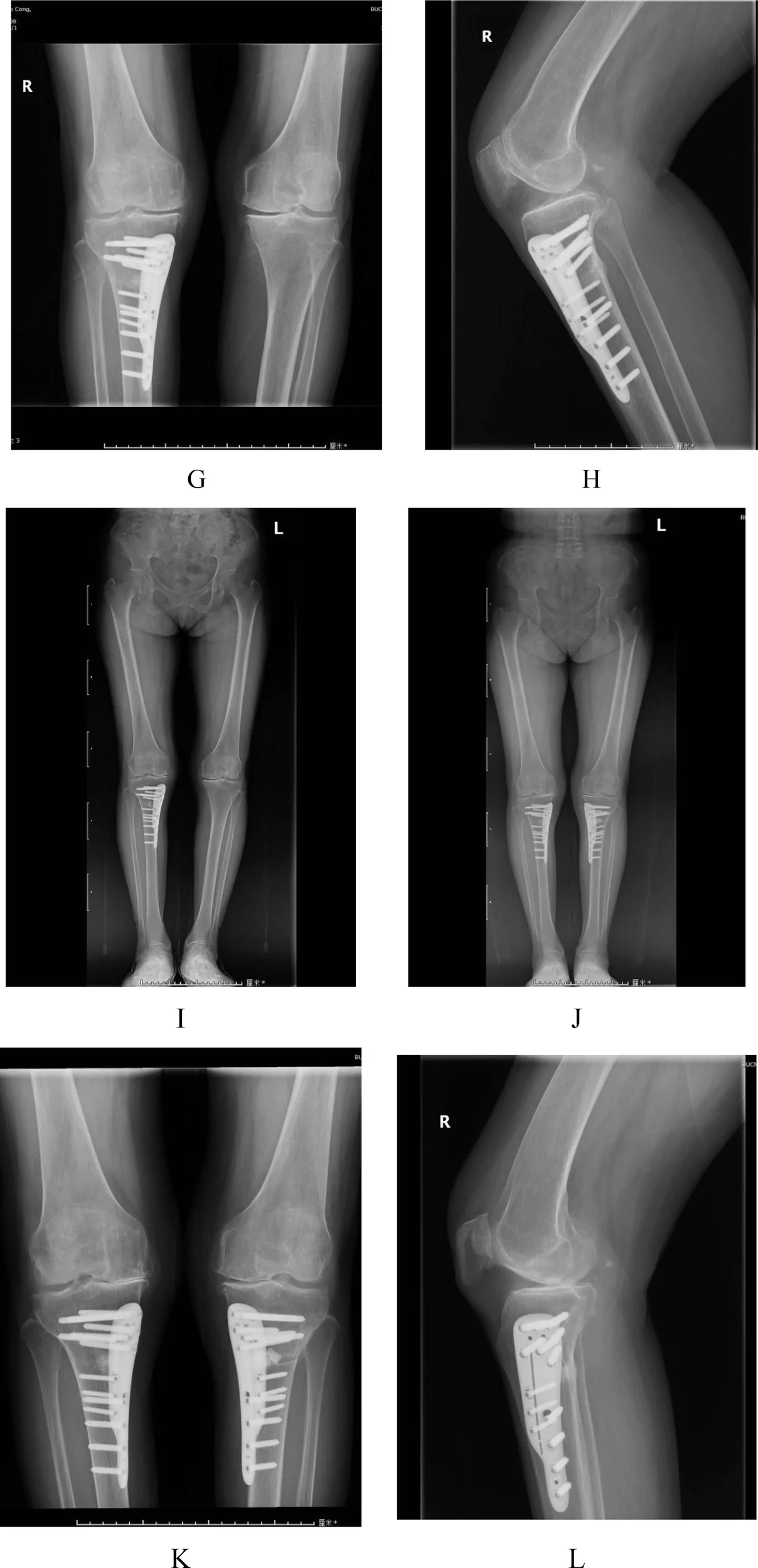
Typical case presentation. **(A–C)** Preoperative full-length lower limb film and AP and lateral views; **(D–F)** Immediate postoperative full-length lower limb film and AP and lateral views; **(G–I)** 1-year postoperative full-length lower limb film and AP and lateral views; **(J–L)** 2-year postoperative full-length lower limb film and AP and lateral views.

## 4 Discussion

In this retrospective cohort, both groups improved after HTO, and no statistically significant between-group differences were detected at 24 months. The estimated differences in VAS, WOMAC, Lysholm, and alignment measures were small, but their 95% confidence intervals quantify uncertainty rather than establish equivalence. The study therefore supports only the narrower conclusion that an association with inferior 24-month outcomes was not detected in this cohort (12–15).

Pain and function improved in both groups, consistent with the expected effect of mechanical-axis correction and postoperative rehabilitation (16–22). Locking-plate fixation and allograft support may also help maintain correction in patients with reduced bone mass. These mechanisms are plausible explanations, but they were not directly tested and should not be interpreted as proof that bone quality is unimportant.

Alignment correction was maintained through 24 months in both groups, and PTS remained stable. The osteopenia/osteoporosis group nevertheless had numerically longer healing time and slightly greater correction loss. Although these differences were not statistically significant, the estimates and low event counts do not exclude clinically meaningful effects on delayed union, fixation failure, or loss of correction (23–26).

The treatment protocol limits interpretation. Every patient in the osteopenia/osteoporosis group received calcium, vitamin D3, and alendronate, whereas the normal-bone-mass group did not. Bone status and treatment were therefore completely confounded, so their independent effects cannot be separated statistically. The present findings apply to the combined care pathway of HTO plus the reported bone-health regimen and neither prove nor refute the need for routine postoperative anti-osteoporosis treatment. A prospective study with standardized or randomized treatment would be required to answer that question (27).

Osteopenia and osteoporosis were combined to preserve sample size, but they represent clinically distinct states. Only 18 patients met the osteoporosis threshold, which would yield imprecise subgroup estimates and unstable adjusted models. We therefore avoid referring to the entire exposure group as osteoporosis and acknowledge that a larger three-group analysis is needed before severity-specific conclusions can be drawn.

Several limitations constrain inference. The retrospective complete-case design is vulnerable to selection bias, no primary outcome or analysis plan was prespecified, no *a priori* sample-size calculation was performed, and multiple exploratory comparisons were reported without multiplicity adjustment. The cohort was small for rare complications, follow-up was limited to 24 months, and radiographic reliability was not formally assessed. Treatment was completely confounded with bone-status group, and the combined osteopenia/osteoporosis analysis prevented severity-specific inference. These limitations mean that non-significant findings cannot exclude clinically meaningful differences or support causal or equivalence claims.

In this retrospective cohort, low bone mass or osteoporosis was not significantly associated with inferior clinical or radiographic outcomes at 24 months after HTO. This finding should not be interpreted as evidence of equivalence or as proof that bone quality has no effect. Treatment was completely confounded with bone-status group, and the study lacked the power to exclude clinically meaningful differences or uncommon complications. Larger prospective studies with prespecified outcomes, severity-specific bone-status groups, standardized treatment protocols, and adequate follow-up are required.

## Data Availability

The raw data supporting the conclusions of this article will be made available by the authors, without undue reservation.
